# Validation of a qualitative real-time PCR assay for the detection of *Candida auris* in hospital inpatient screening

**DOI:** 10.1128/jcm.00158-24

**Published:** 2024-05-01

**Authors:** Lauren C. Franco, Mahmoud Ahmed, Christopher G. Kendra, R. Matthew Sperling, Kayla Van Benten, John-Paul Lavik, Christopher L. Emery, Ryan F. Relich, Kenneth Gavina

**Affiliations:** 1 Department of Pathology and Laboratory Medicine, Indiana University School of Medicine, Indianapolis, Indiana, USA; 2 Division of Clinical Microbiology, Indiana University Health, Indianapolis, Indiana, USA; University of Utah, Salt Lake City, Utah, USA

**Keywords:** mycology, infectious disease, hospital infections, *Candida auris*, molecular methods, public health

## Abstract

*Candida auris* is a multidrug-resistant opportunistic fungal pathogen capable of causing serious infections and healthcare-associated outbreaks. Screening for colonization with *C. auris* has become routine and is recommended in many hospitals and healthcare facilities as an infection control and prevention strategy. Subsequently, and since there are currently no FDA-approved tests for this purpose, clinical microbiology laboratories have become responsible for developing protocols to detect *C. auris* using axial and inguinal screening swabs. In a College of American Pathologists-accredited large academic healthcare center setting, we implemented a laboratory-developed nucleic-acid amplification test for the detection of *C. auris* DNA. Our test validation evaluated the performance of the DiaSorin *C. auris* primer set used in a real-time qualitative PCR assay on the LIAISON MDX thermocycler with the Simplexa Universal Disc. The assay was highly sensitive and specific, with a limit of detection of 1–2 CFU/reaction, with no observed cross-reactivity with other *Candida* spp., bacterial skin commensal organisms or commonly encountered viruses. When run in parallel with a culture-based detection method, the PCR assay was 100% sensitive and specific. The assay was precise, with low variability between replicates within and between runs. Lastly, pre-analytical factors, including swab storage time, temperature, and transport media, were assessed and found to have no significant effect on the detection of *C. auris* at variable concentrations. Taken together, this study expands the available options for nucleic acid detection of *C. auris* and characterizes pre-analytical factors for implementation in both high- and low-volume laboratory settings.

**IMPORTANCE:**

This study overviews the validation and implementation of a molecular screening tool for the detection of *Candida auris* in a College of American Pathologist-accredited clinical laboratory. This molecular laboratory-developed test is both highly sensitive and specific and has significant health-system cost-savings associated with significantly reduced turn-around-time compared to traditional standard-of-care culture-based work up. This method and workflow is of interest to support clinical microbiology diagnostics and to help aid in hospital inpatient, and infection prevention control screening.

## INTRODUCTION

*Candida auris* is an opportunistic fungal pathogen that has impacted healthcare and long-term care (LTC) facilities worldwide due to its ability to cause severe infections in elderly and immunocompromised populations. Originally isolated in 2009 from a human ear canal in Japan, the prevalence of *C. auris* has increased, with 3,270 clinical infections and 7,413 positive screens reported from 2019 to 2021 in the United States (1, 2). Hallmark characteristics of *C. auris*, which set it apart from other pathogenic *Candida* spp., include the ability of *C. auris* to colonize skin, form persistent, disinfectant-resistant biofilms on healthcare surfaces, and rapidly develop resistance to multiple classes of antifungal agents. Together, these characteristics advantageously contribute to the organism’s ability to inhabit and spread in both healthcare and LTC facilities.

Skin colonization represents the largest outbreak risk to clinical environments as asymptomatic carriers of *C. auris* can contaminate high-touch surfaces and be transferred to other susceptible non-carriers (3). Additionally, in environments where *C. auris* enters a biofilm state, these pathogens resist common disinfectants, including chlorhexidine, sodium hypochlorite, peracetic acid, and caspofungin (4, 5). Given these factors, implementation of initiatives that aid identifying patient reservoirs of *C. auris* and engaging the appropriate infection prevention methods to control additional dispersal of this pathogen have become necessary. To combat the spread of *C. auris* in healthcare and LTC facilities, screening patients for *C. auris* colonization has become routine. Culture-based screening of patient axilla and inguinal swabs involves manual interpretation of media which select for and differentiate *Candida* spp. after 18–36 h of incubation (6–8). While reliable, this form of screening can create a large burden for clinical laboratories when screening all inpatient admissions.

Nucleic acid testing for *C. auris* offers a more directed approach with faster turn-around for patient screening. While there is no FDA-approved molecular assay for *C. auris* screening currently available, a validated form of TaqMan real-time PCR (rt-PCR) has been developed by the Wadsworth Center in New York that utilizes the BD MAX platform to analyze extracted nucleic acids from swab specimens (9, 10). Other commercially available nucleic acid tests, including the OLM Diagnostics AurisID, BioGX *Candida auris,* and Fungiplex *Candida auris*, have been developed and are indicated for research use only within the United States. These tests use variously sourced materials for the detection of *C. auris* and have variable specificities and limits of detection (11).

In this work, we validated a *C. auris* nucleic acid test using the DiaSorin *C. auris* primer set on the DiaSorin LIAISON MDX and DiaSorin Simplexa Universal Disc platform to create a direct-from-swab screening approach. Additionally, to demonstrate the robustness of this screening assay in operational scaling from daily runs to weekly pooled testing, we assessed the stability of mocked biological swabs stored at both ambient and refrigerated temperatures over the course of one week. Taken together, this work demonstrates a culture-free, highly sensitive and specific *C. auris* screening approach that can be integrated into both low- and high-volume laboratory operations while meeting minimal requirements for both the Clinical Laboratory Improvement Amendments (CLIA) and the College of American Pathologists (CAP).

## MATERIALS AND METHODS

### Patient samples and controls

Clinical specimens were collected using BBL CultureSwabs in liquid Stuart or liquid Amies medium (Becton Dickinson, Franklin Lakes, NJ) or Transystem swabs in liquid Stuart medium (Copan, Murrieta, CA). Clinical specimens were collected for *C. auris* colonization screening by swabbing a combination of axial, inguinal, and/or other external body sites. Clinical swab samples were inoculated to Sabouraud dextrose agar with chloramphenicol (Remel, San Diego, CA) for downstream identification via MALDI-TOF and Candida CHROMagar (Hardy Diagnostics, Springboro, OH) plates for culture-based *C. auris* screening according to the method developed by the U.S. Centers for Disease Control and Prevention (12). The same swabs were then processed for detection by PCR via the method described below. A total of 282 patient samples were enrolled for this study. *C. auris* positive controls were made from a stock of *C. auris* (MYA-5002; ATCC, Manassas, VA). This stock culture was quantified by viable plate count, diluted to a working concentration of 5 × 10^5^ CFU/mL, and frozen at −80°C. All samples were prepared and manipulated within a biological safety cabinet in a BSL-2 laboratory with enhanced precautions.

### Specimen preparation and heat lysis

Swabs were broken off into a 5 mL tube containing 500 µL of Tris-EDTA (TE) pH 8.0 and vortexed. Five microliters of Fungal Lysis Solution (DiaSorin, Cypress, CA) and 50 µL of the sample-containing TE buffer were added to a 2-mL screwcap tube and vortexed. This mixture was then incubated at 60 ± 2°C in a rocking incubator at 25 strokes/minute for 30 minutes. A *C. auris* positive control and no template control (NTC) were also prepared and lysed with the patient samples. If testing was to be performed within 24 hours of lysis, lysates were stored at 2–8°C. If testing was to be performed beyond 24 hours of lysis, lysates were stored at −80°C.

### Qualitative real-time PCR

A PCR master mix was prepared using temperature-activated (TA) master mix (DiaSorin, Cypress CA), *C. auris* primer pair (DiaSorin, Cypress, CA), Simplexa extraction and amplification control set (DiaSorin, Cypress, CA), and DNAse-free H_2_O. For each sample, 4 µL of TA Master Mix, 3.25 µL of DNAse free H_2_O, 0.40 µL of internal control DNA, 0.20 µL of *C. auris* Primer Pair, and 0.15 µL of internal control primer were combined to form the master mix. The master mix was briefly vortexed and centrifuged for homogeneity. Eight microliters of master mix and 2 µL of sample or control were aliquoted into each well of a Simplexa Universal Disc (DiaSorin, Cypress, CA). The Universal Disc was loaded on the LIAISON MDX thermocycler (DiaSorin, Cypress, CA) and run according to the following protocol: 10 minute initial activation step at 97°C, followed by 45 cycles of 10 second denaturation at 97°C and 30 seconds annealing at 60°C. Samples with a Ct value less than 40 were considered positive.

### Sensitivity, specificity, and reproducibility

Analytical sensitivity was determined by creating a serial dilution of *C. auris* (MYA-5002; ATCC, Manassas, VA) from 10^7^ to 10^2^ CFU/mL as quantitated by viable plate count. The limit of detection (LOD) was determined by probit analysis with a 95% CI. Analytical precision was assessed by measuring the coefficient of variation (%CV) in triplicate of quantitated *C. auris* controls. Analytical specificity was determined by assessing microorganisms and viruses obtained from a combination of clinical specimens and reference samples (ATCC, Manassas VA; Zeptometrix, Franklin MA) at concentrations of 10^5^ CFU/mL yeast and bacteria, 10^5^ plaque forming units (PFU)/mL, TCID_50_/mL, or copies/mL for viruses. Yeast and bacteria were quantified by viable plate count and diluted to a final concentration using sterile saline. Viruses were obtained pre-titred and diluted to a final working concentration using sterile phosphate-buffered saline (PBS). Axial swabs from confirmed *C. auris*-negative volunteers were then inoculated with the non-specific analytes of interested and screened by the above-described methodology.

Clinical sensitivity, specificity, positive predictive value (PPV), and negative predictive value (NPV) were determined by assessing 282 clinical specimens by both the real-time PCR assay and fungal culture as the gold standard comparator. Clinical specimens were plated on Sabouraud dextrose agar with chloramphenicol and HardyCHROM Candida agar plates.

### Sample stability

To assess stability of the samples over time and in different transport media, mock high (10^7^ CFU/mL) and low (10^4^ CFU/mL) samples were created and stored in Liquid Stuarts, Liquid Amies, and Gel Amies transport media that contained axial swabs from people proven to be *C. auris*-negative. A subset of samples was tested immediately (Day 0), while the others were stored at 4°C or room temperature and tested on days 1, 2, 3, and 7.

### Statistics

LOD was determined by probit analysis using MedCalc version 20.113 (MedCalc Software, Ostend, Belgium). For the sample stability analyses, one-way analysis of variance (ANOVA) was performed and visualized in Prism version 9.2.0 (Graphpad, Boston, MA).

## RESULTS

### Analytical sensitivity, specificity, and precision

**Fig 1:**
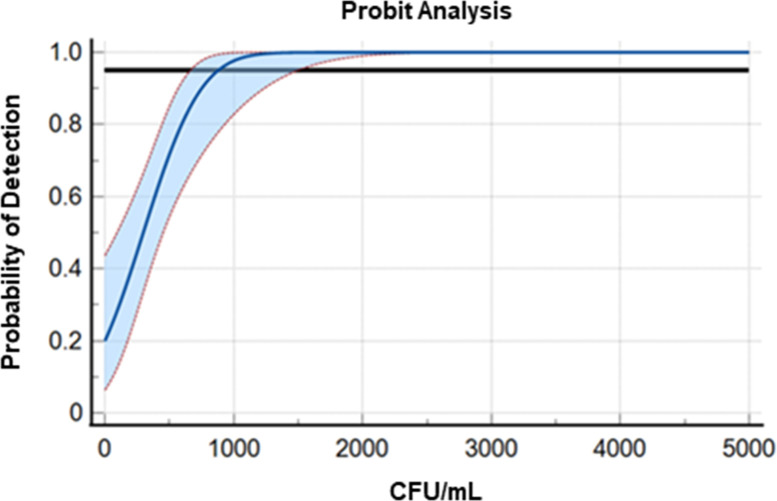
LOD as determined by probit regression for *Candida auris*. The black horizontal line represents 0.95 probability, the blue line depicts the regression curve, and the red dotted line represents the 95% confidence intervals.

Analytical sensitivity and precision were determined by assaying a dilution series ranging from 10^7^ to 10^2^ CFU/mL *C*. *auris* that was quantified by viable plate count. Analytical sensitivity of the assay, as determined by probit analysis is 879.5 CFU/mL with a 95% CI from 66.31 to 1497.51 CFU/mL (Fig. 1). This equates to 1–2 CFU/reaction, which is consistent with other published laboratory developed tests (LDTs) for *C. auris* screening from surveillance swabs via real-time PCR (9, 10, 13). The dilution series was performed in triplicate to determine precision (Table 1), which was measured by the coefficient of variance (%CV) of the generated *C*_t_ values. For samples run on the same disk on the same day (intra-assay variation), %CV ranged from 0.56% to 4.94%. For samples run on different discs (inter-assay variation), %CV ranged from 0.60% to 7.90%. Analytical specificity was determined by performing the assay with no template controls (NTC), other *Candida* spp., skin commensal bacteria, and frequently encountered viruses. No amplification or cross-reactivity was observed with the NTC, microorganisms, or viruses tested, but the internal control was detected in each reaction (Table 2).

**TABLE 1:** Reproducibility and repeatability of the *C. auris* real-time PCR assay^*a*^

*C. auris* dilution	Intra-assay mean (Ct value)	%CV	Inter-assay mean (Ct value)	%CV
1 × 10^7^ CFU/mL	15.08	1.64	15.36	7.90
1 × 10^6^ CFU/mL	22.48	0.59	22.00	3.19
1 × 10^5^ CFU/mL	26.42	0.56	26.33	0.60
1 × 10^4^ CFU/mL	30.22	1.21	30.43	1.26
1 × 10^3^ CFU/mL	34.38	5.91	34.35	4.87
5 × 10^2^ CFU/mL	36.43	4.94	36.43	4.94
1 × 10^2^ CFU/mL	38.60	1.60	38.60	1.60

^*a*^ Ct, cycle threshold; %CV, coefficient of variation; CFU, colony-forming unit.

**TABLE 2:** Analytical specificity was determined by screening for *C. auris* in specimens containing other *Candida* spp., bacterial skin flora, and clinically relevant viruses

Other organisms tested		
*C. glabrata*	*E. coli*	Mumps
*C. albicans*	*Enterococcus faecalis*	*Neisseria gonorrhoeae*
*C. doubushaemulonii*	Epstein Barr virus	*Neisseria meningitidis*
*C. dubliniensis*	Enterovirus 71	*Pseudomonas aeruginosa*
*C. haemulonii*	Influenza virus A H1 & H3, B	Parvovirus B19
*C. kefyr*	*Gardnerella vaginalis*	Parainfluenza virus 1, 2, 4A & 4B
*C. krusei*	Human herpes virus 6 & 7	Rhinovirus A
*C. lusitaniae*	Human papilloma virus 16 & 18	Respiratory syncytial virus A
*C. parapsilosis*	Herpes simplex virus 1 & 2	Rubella
*C. tropicalis*	*Klebsiella pneumoniae*	*Staphylococcus aureus*
Adenovirus	*Moraxella catarrhalis*	*Staphylococcus epidermidis*
*Cutibacterium acnes*	*Micrococcus luteus*	*Staphylococcus saprophytius*
Cytomegalovirus	*Mycoplasma pneumonia*	Viridans streptococci
*Corynebacterium* spp.	*Mycoplasma genitalium*	Varicella zoster virus
Coxsackie virus A16	Measles	

### Sample stability

**Fig 2:**
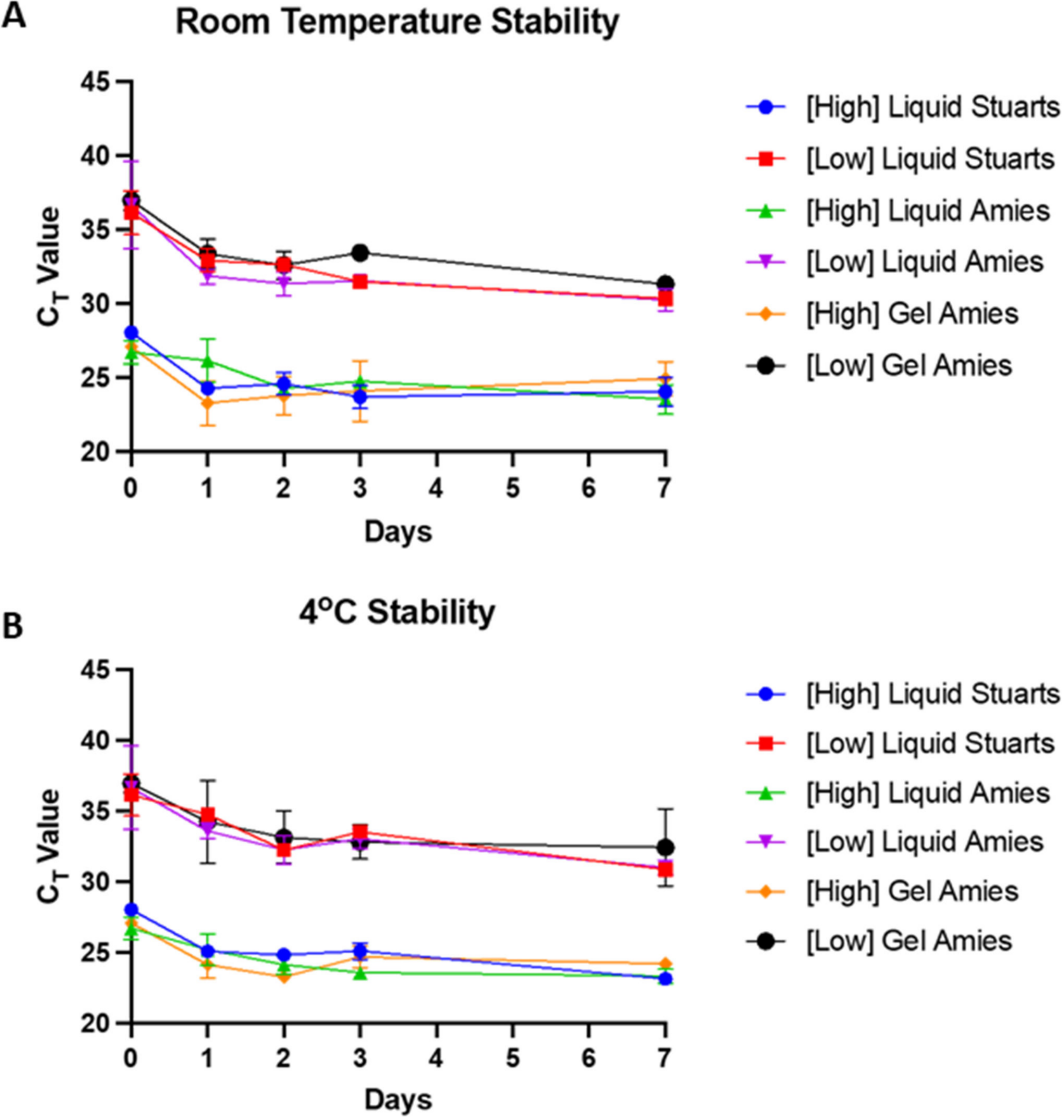
Sample stability over time at room-temperature (**A**) and 4°C (**B**) in Amies and Stuart’s transport media.

Sample stability over time, at different holding temperatures, and in different transport media were assessed to determine the effect these pre-analytical factors had on the assay results. No significant differences were observed between day 0 and day 7, at room temperature or at 4°C, across the different tested transport media (Fig. 2). At room temperature, Ct values ranged from 23.3 to 26.1 and 30.2 to 35.3 for the high-concentration and low-concentration sample swabs (Amies and Stuarts), respectively. At 4°C, Ct values ranged from 23.1 to 25.1 and 30.9 to 35.3 for the high concentration and low concentration sample swabs (Amies and Stuarts), respectively.

### Clinical sensitivity and specificity relative to culture

Clinical sensitivity, specificity, positive predictive value (PPV), and negative predictive value (NPV) were determined by assessing 282 clinical specimens by both the qualitative real-time PCR assay and fungal culture as the gold standard comparator. *C. auris* was detected by both conventional culture and rt-PCR assay in 23 total clinical samples, for a 100% clinical sensitivity rate. *C. auris* was not detected by culture or rt-PCR in 259 samples, for a 100% clinical specificity rate. Positive predictive value and NPV were also 100% (Table 3).

**TABLE 3:** Performance of the *C. auris* real-time PCR assay compared to culture as gold standard testing

		Expected result	
		Positive	Negative
rt-PCR result	Positive	23	0
	Negative	0	259
Clinical performance		Value	
Sensitivity		100.0%	
Specificity		100.0%	
Positive predictive value		100.0%	
Negative predictive value		100.0%	

## DISCUSSION

Since the emergence of *C. auris*, and subsequent implementation of infection control and prevention measures, screening of patients for *C. auris* colonization has created a new demand for clinical microbiology laboratories. While culture-based methods of detection are acceptable, the turn-around-time (TAT) from sample collection to result is 1–5 days, potentially delaying patient transfer to another unit or facility, or use of inappropriate contact precautions. Molecular assays are well-suited for screening tests such as this, as they can be scaled to handle large volumes, provide accurate results with a decreased TAT, and reduce overall healthcare spending. Although molecular tests are generally more expensive than culture-based tests, the decrease in TAT and subsequent reduction in hospital stay when a negative *C. auris* screen is required prior to discharge to a LTC facilities justify the more expensive test for an overall cost savings.

Without available FDA-approved molecular assays for *C. auris* detection, clinical laboratories must perform studies on LDTs to assess their performance if they wish to bring on testing. Minimum criteria that must be assessed in such a validation include analytical sensitivity and specificity, precision, LOD, and comparison to a gold standard method, as defined by both CAP and CLIA. In this validation study, these parameters were consistent with, or improved, compared to other similar studies. Previously published validations have shown sensitivity ranging from 89% to 100% and specificity ranging from 92% to 100% (9, 10, 13, 14). This assay demonstrated equivalent, or superior, sensitivity and specificity with both metrics at 100%. It is worth noting that the inter-assay %CV was determined to be 7.9% at a concentration of 10^7^ CFU/mL, which can be concerning, particularly at higher concentrations. This was due to a single replicate (*n* = 15 total replicates per dilution) resulting in a Ct value of 19.5, which may be attributed to the inherent small volume handling of the DiaSorin assay. When this value is omitted, the adjusted calculated %CV was determined to be 2.7%. No cross-reactivity was observed with other *Candida* spp., including *C. haemulonii* and *C. doubushaemulonii,* which are closely related to *C. auris* and have had misidentifications documented (15, 16). Additionally, no cross-reactivity was observed with bacterial skin commensal organisms, or with viruses and bacteria commonly encountered in clinical specimens, indicating the primers are highly specific for *C. auris*.

After evaluating the performance of the assay, we assessed sample stability over time, at two different temperatures, in different transport media, and at high and low analyte concentrations. These pre-analytical factors are important to evaluate for this test to withstand unexpected delays in testing and to allow for batching of samples. No changes in Ct values were observed between day 0 and day 7 in any of the conditions tested, indicating that samples can be stored for at least a week before testing, if necessary. Furthermore, samples can be stored at room temperature or 4°C without affecting detection. The sample stability was consistent in all three transport media, providing greater flexibility for sample collection.

Replacing traditional culture with nucleic acid testing for *C. auris* carrier screening reduces a large labor burden to clinical laboratories by automating batch testing of samples. The robustness and commonplace nature of nucleic acid testing enables laboratories in sizes ranging from small community hospitals up to centralized clinical pathology facilities to bring this test in-house. One limitation of this type of testing compared to culture-based methodology is the analytical endpoint only assesses the presence or absence of the yeast and does not yield an isolate that could be used if *C. auris* antifungal susceptibility testing or outbreak tracing via whole-genome sequencing is necessary. A limitation of this study is the small number (*n* = 23) of positive clinical samples that were collected over the study period. A larger number of positive samples could impact the predictive values associated with this assay and increase the probability of detecting errors resulting in false negative or false positive results. Furthermore, at the time of study, CFU counts of the PCR-positive specimens were not performed.

This study assessed the performance of a commercially available primer set and reagents implemented for use on a commercially available platform. Other clinical microbiology laboratories could purchase these reagents and instruments and replicate this process to validate this assay in their own laboratories. Furthermore, pre-analytical factors were tested and shown not to have a significant influence on sample stability and downstream assay performance, increasing the utility and robust nature of this assay. Ultimately, this work addresses a pressing need for rapid, high-throughput testing options for *C. auris* colonization screening.
